# *RhMKK9*, a rose MAP KINASE KINASE gene, is involved in rehydration-triggered ethylene production in rose gynoecia

**DOI:** 10.1186/s12870-017-0999-1

**Published:** 2017-02-23

**Authors:** Jiwei Chen, Qian Zhang, Qigang Wang, Ming Feng, Yang Li, Yonglu Meng, Yi Zhang, Guoqin Liu, Zhimin Ma, Hongzhi Wu, Junping Gao, Nan Ma

**Affiliations:** 1 Department of Ornamental Horticulture, China Agricultural University, Beijing, 100193 China; 2 Beijing Key Laboratory of Development and Quality Control of Ornamental Crops, China Agricultural University, No. 2 Yuanmingyuan West Road, Haidian District, Beijing, 100193 China; 3 Flower Research Institute, Yunnan Academy of Agricultural Sciences, Kunming, 650205 China; 4 College of Biology and Environmental Engineering, Provincial Key Laboratory of Biocontrol, Guiyang University, Guiyang, 550005 China; 5 College of Horticulture and Landscape, University, Yunnan Agricultural University, Kunming, 650201 China

**Keywords:** Rose flower, Gynoecia, *RhMKK9*, Rehydration, Ethylene biosynthesis, DNA methylation

## Abstract

**Background:**

Flower opening is an important process in the life cycle of flowering plants and is influenced by various endogenous and environmental factors. Our previous work demonstrated that rose (*Rosa hybrida*) flowers are highly sensitive to dehydration during flower opening and the water recovery process after dehydration induced ethylene production rapidly in flower gynoecia. In addition, this temporal- and spatial-specific ethylene production is attributed to a transient but robust activation of the rose MAP KINASE6-ACC SYNTHASE1 (RhMPK6-RhACS1) cascade in gynoecia. However, the upstream component of RhMPK6-RhACS1 is unknown, although RhMKK9 (MAP KINASE KINASE9), a rose homologue of *Arabidopsis* MKK9, could activate RhMPK6 in vitro. In this study, we monitored *RhMKK2/4/5/9* expression, the potential upstream kinase to RhMPK6, in rose gynoecia during dehydration and rehydration.

**Results:**

We found only *RhMKK9* was rapidly and strongly induced by rehydration. Silencing of *RhMKK9* significantly decreased rehydration-triggered ethylene production. Consistently, the expression of several ethylene-responsive genes was down regulated in the petals of *RhMKK9*-silenced flowers. Moreover, we detected the DNA methylation level in the promoter and gene body of *RhMKK9* by Chop-PCR. The results showed that rehydration specifically elevated the DNA methylation level on the *RhMKK9* gene body, whereas it resulted in hypomethylation in its promoter.

**Conclusions:**

Our results showed that RhMKK9 possibly acts as the upstream component of the RhMKK9-RhMPK6-RhACS1 cascade and is responsible for water recovery-triggered ethylene production in rose gynoecia, and epigenetic DNA methylation is involved in the regulation of *RhMKK9* expression by rehydration.

**Electronic supplementary material:**

The online version of this article (doi:10.1186/s12870-017-0999-1) contains supplementary material, which is available to authorized users.

## Background

Plants are exposed to various abiotic and biotic stresses because of their sessile life style. Therefore, for improved survival, plants develop a system that can rapidly sense signals from a changing environment and respond adaptively and/or defensively by modulating the internal physiological, biochemical, and molecular processes [1, 2]. For most crops, water deficit causes a major limitation to the yield. It has been well documented that water deficit results in several physiological changes, including wilting, stomatal closure, hormone imbalance, and suppression of cell growth and photosynthesis [3, 4]. After dehydration, plants are able to recover quickly once water is available again via the rehydration process [5–7].

Although signal reception and the transduction pathway of dehydration have been extensively reported, they remain largely unknown for the rehydration process. Using a DNA microarray, Oono et al. [5] found that rehydration-responsive genes included genes related to both stressed status release and growth recovery in *Arabidopsis*. In addition, ethylene-biosynthetic genes and genes responsive to jasmonic acid, gibberellin, and auxin were activated by rehydration, suggesting hormone balance is involved in the water recovery of plants.

In plants, the mitogen-activated protein kinase (MAPK) cascade plays an essential role in the signaling pathway of multiple abiotic and biotic stress cues [1, 2, 8–11]. The MAPK cascade is initiated by a mitogen-activated protein kinase kinase kinase (MAPKKK, MAP3K, or MEKK), which reversibly phosphorylates a mitogen-activated protein kinase kinase (MAPKK, MAP2K, or MKK) and subsequently phosphorylates the mitogen-activated protein kinase (MAPK or MPK). Then, MPK phosphorylates its downstream target proteins to modulate various developmental and physiological processes [12]. In *Arabidopsis*, several components of the MAPK cascade, such as MPK4 and MPK6, were reported to be activated by drought [13, 14]; a recent report showed that MPK6 could be activated by drought, but inactivated by rehydration in *Arabidopsis* seedlings [15].

Cut roses (*Rosa hybrida*) are an important ornamental crop globally. Dehydration is a considerable postharvest problem for cut roses because their market supply is highly dependent on long-distance transportation, resulting in severe dehydration for this duration. Recently, we reported that in rose (*R. hybrida*) flowers, rehydration following dehydration triggered rapid, but transient, ethylene production in the gynoecia, namely all the carpels and pistils in a flower [7]. During dehydration and rehydration, the protein level of a specific MAP kinase, RhMPK6, was maintained at a constant high level. However, RhMPK6 activity was only detected within 1 h of rehydration [16]. Furthermore, activated RhMPK6 phosphorylated and stabilised RhACS1, a rate-limiting enzyme of ethylene biosynthesis, and resulted in an ethylene burst in gynoecia. Ethylene plays an important role in flower opening and senescence in roses [17–20]; thus, we speculated that the RhMPK6-RhACS1 module might be crucial to sense the rehydration signal and transmit it to ethylene to regulate flower opening and senescence in roses.

Previously, we found that a MAPKK protein, RhMKK9, could phosphorylate the RhMPK6 protein in vitro [16]. However, to date it is unknown whether RhMKK9 is the actual upstream component activating RhMPK6 in gynoecia during rehydration. In the present study, we isolated the possible MAPK kinases upstream from RhMPK6 from roses and monitored their expression pattern during dehydration and rehydration. We found that *RhMKK9* is the specific MAPK kinase that phosphorylates RhMPK6 and is responsible for the rehydration-induced ethylene production in gynoecia. Furthermore, methylation-sensitive PCR showed that DNA methylation of the promoter of *RhMKK9* contributes to rehydration-induced upregulation of *RhMKK9* expression.

## Results

### *RhMKK9* expression is rapidly and strongly induced by dehydration in rose gynoecia

We previously reported that ethylene production could be rapidly but transiently induced by rehydration in rose gynoecia when the flowers were subjected to dehydration-rehydration treatment [7]. Moreover, temporal- and spatial-specific activation of an RhMPK6-RhACS1 cascade is responsible for this rehydration-induced ethylene production [16].

**Fig. 1:**
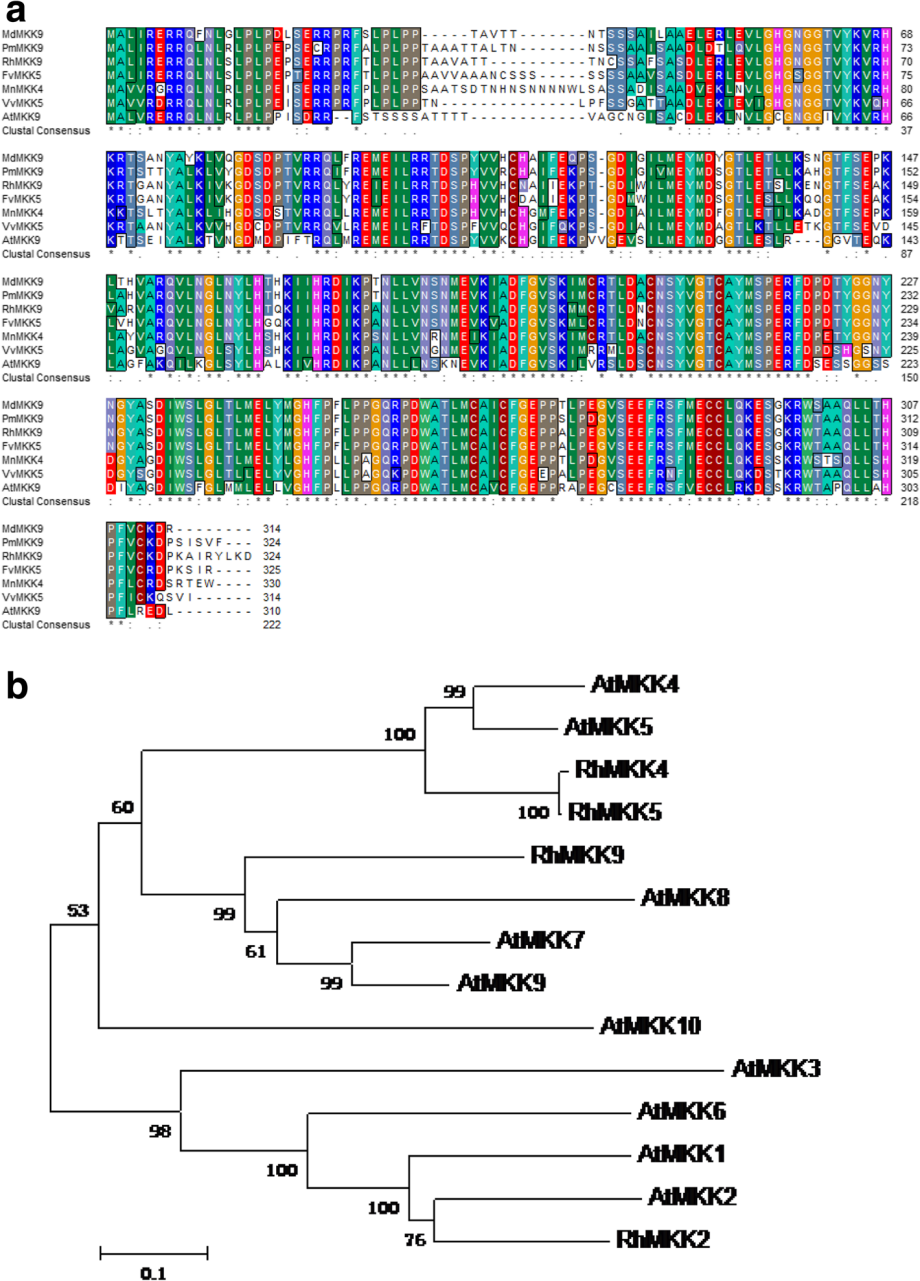
Alignment (**a**) and phylogenetic tree assay (**b**) of RhMKK9. A phylogenetic tree was constructed using the predicted RhMKK2/4/5/9 amino acid sequences and MKK homologues from various plant species. The tree was constructed using MEGA 5.2. Bootstrap values indicate the divergence of each branch and the scale bar represents the branch length at 0.1 substitutions per site

In *Arabidopsis*, MPK6 could be activated by MAPK kinases MKK2/4/5/9 [21]. Thus, we searched the rose homologues of *AtMKK2*, *AtMKK4*, *AtMKK5*, and *AtMKK9* in a rose transcriptome database [22], and cloned their full-length sequences. We designated these sequences as *RhMKK2* (*RU26187*, GenBank Acc. No. KP269070), *RhMKK4* (*RU27106*, GenBank Acc. No. KP269071), *RhMKK5* (*RU11454*, GenBank Acc. No. KP269072), and *RhMKK9* (*RU24635*, GenBank Acc. No. KP269073), respectively (Fig. 1; Additional file 1: Figure S1). The full-length cDNA of *RhMKK9* is 1,367 bp and contains a 975-bp open reading frame flanking with a 59-bp 5′-untranslated region (UTR) and a 333-bp 3′-UTR. For *RhMKK2*, *RhMKK4*, and *RhMKK5*, the cDNA full-length was 1453, 1522, and 1508 bp, respectively (Additional file 1: Table S1).

**Fig. 2:**
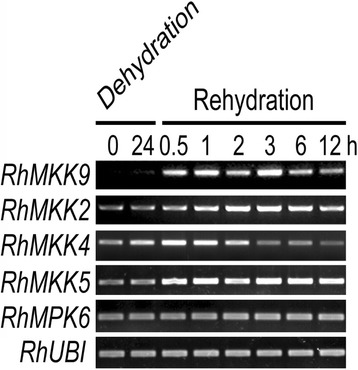
Expression pattern of *RhMKK2*, *RhMKK4*, *RhMKK5*, *RhMKK9*, and *RhMPK6* in gynoecia of rose flowers during dehydration and rehydration processes. *RhUBI* was used as an internal control. Three biological replicates were tested for each time point and representative results were demonstrated

To identify which MKK gene was the major contributor to rehydration-induced ethylene biosynthesis in rose gynoecia, we firstly detected the expression level of all four genes in response to dehydration and rehydration in gynoecia. *RhMKK4* expression was induced by 24 h dehydration in gynoecia, whereas expression of *RhMKK2* and *RhMKK9* was almost not affected. When the flowers were subjected to rehydration, *RhMKK2/4/5* transcription appeared to be slightly induced 30 min after rehydration. However, *RhMKK9* expression was rapidly and strongly induced within 30 min after rehydration. After 12 h of rehydration, *RhMKK9* transcription decreased to a relative low level (Fig. 2). Meanwhile, *RhMPK6* expression was retained at a relatively constant level during dehydration and rehydration, consistent with RhMPK6 protein accumulation in our previous report [16]. Furthermore, rehydration-induced activation of RhMPK6-RhACS1 and consequently ethylene biosynthesis occurred during 0.5–1 h of rehydration and then dropped quickly [16]. According to the timing of the expression, we speculated that RhMKK9 might be the MAPK kinase responsible for the activation of the RhMPK6-RhACS1 cascade in rose gynoecia.

### Silencing of *RhMKK9* suppressed ethylene production and petal senescence in rose flowers after dehydration and rehydration treatment

**Fig. 3:**
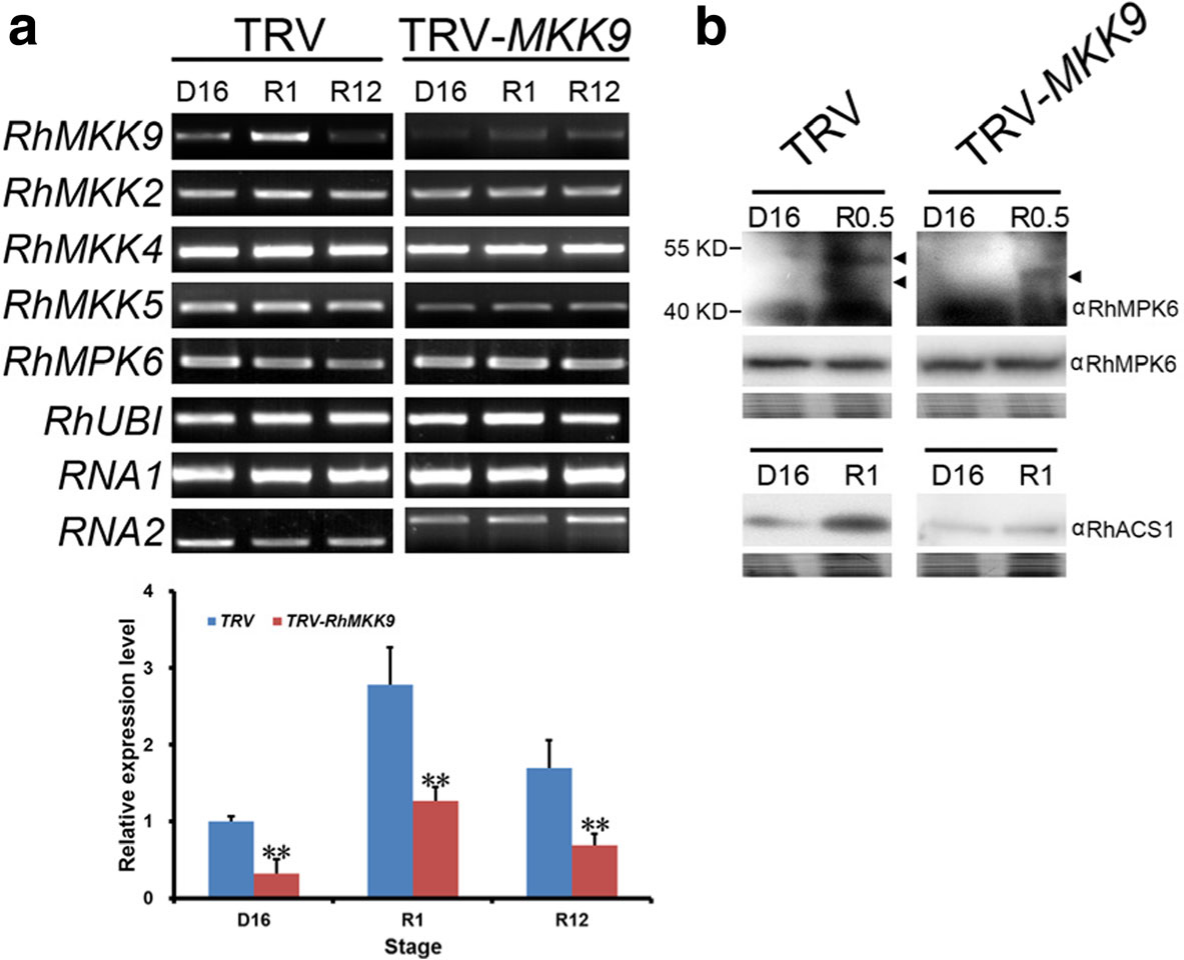
Effects of *RhMKK9* silencing in rose flowers. **a** Expression pattern of *RhMKKs* and *RhMPK6. Upper panel*, semi-quantitative RT-PCR assay of *RhMKK9*, *RhMKK2*, *RhMKK4*, *RhMKK5*, and *RhMPK6* in TRV- and TRV-*MKK9*-infected flowers. *RhUBI* was used as an internal control. *Bottom panel,* quantification of *RhMKK9* expression. At least three biological replicates were tested for each time point. Asterisks in the bottom panel indicated significant differences calculated using the *t* test (**p < 0.01). **b** The RhMPK6 phosphorylation level and RhMPK6 and RhACS1 protein levels in TRV- and TRV-*MKK9*-infected flowers. The RhMPK6 phosphorylation level was monitored using the Phos-tag reagent. A Coomassie Brilliant Blue-stained blot is shown below to confirm equivalent sample loading. D16, dehydration for 16 h; R0.5, R1, and R12, rehydration for 0.5, 1, and 12 h, respectively

To further confirm the potential role of *RhMKK9* in rehydration-induced ethylene production in gynoecia, we silenced *RhMKK9* -using a virus-induced gene silencing (VIGS) approach. An *RhMKK9*-specific fragment was chosen to construct the TRV2-*MKK9* vector to avoid cross silencing of other MKK genes. We screened positive *RhMKK9*-silenced flowers by detecting RNA1, RNA2, and *RhMKK9* transcription. Both RNA1 and RNA2 transcripts were detected in ~28% flowers, indicating that TRV successfully infected these flowers. RT-PCR analysis revealed that *RhMKK9* expression was significantly reduced in gynoecia of *RhMKK9*-silenced flowers than those of the TRV2 controls, especially at 1 h after rehydration treatment (Fig. 3a). In addition, we detected *RhMKK2/4/5* expression in *RhMKK9*-silenced flowers to confirm whether their expression was affected by silencing *RhMKK9*. As expected, *RhMKK2/4* expression in *RhMKK9*-silenced samples was similar to that in the TRV2 controls (Fig. 3a), indicating that silencing of *RhMKK9* did not influence the transcript accumulation of *RhMKK2/4*. Interestingly, *RhMKK5* transcription appeared to reduce in the silenced flowers relative to the controls. Because *RhMKK9* shared 54% homology of the nucleotide sequence with *RhMKK5*, we assumed that the relatively low level of *RhMKK5* should not result from the *RhMKK9* fragment triggered cross silencing, which requires a high nucleotide sequence homology (>90%).

In addition, we monitored the accumulation pattern of *RhMPK6* transcript and protein, as well as the kinase activity of RhMPK6 in *RhMKK9*-silenced flowers. We tested the RhMPK6 activity and protein level after 30 min of rehydration and RhACS1 protein level after 1 h of rehydration, respectively. This was based on the results from our previous study, which showed that the highest level of RhMPK6 activity and RhACS1 protein appeared at 30 min and 1 h of rehydration, respectively [16]. As expected, the transcript and protein level of RhMPK6 were not affected by *RhMKK9* silencing. However, phos-tag SDS-PAGE demonstrated that *RhMKK9* silencing largely weakened RhMPK6 phosphorylation during rehydration (Fig. 3b). Thus, we considered that low level of RhMPK6 activity caused low level of RhACS1 protein and consequentially low ethylene production in *RhMKK9*-silenced flowers during rehydration. Interestingly, we also detected a weak level of RhMPK6 kinase activity in RhMKK9-silenced lines. This could be attributed to the activity of residual RhMKK9 kinase because the *RhMKK9*-silenced lines were knock-down instead of knock-out lines. These results confirmed that *RhMKK9* functioned upstream from RhMPK6 during rehydration.

**Fig. 4:**
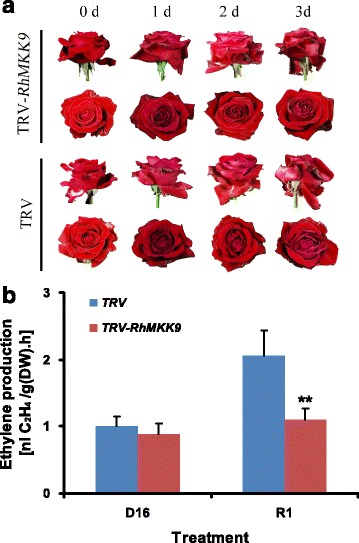
Phenotype (**a**) and ethylene production in gynoecia (**b**) of TRV control and RhMKK9-silenced flowers. At least 15 flowers were used for each time point. Representative results are shown. Asterisks indicated significant differences calculated using the *t* test (**p < 0.01). D16, dehydration for 16 h; R1, rehydration for 1 h. 0, 1, 2, and 3 d, the day(s) after rehydration. Note, compared with the RhMKK9-silenced flower, the TRV flower showed obvious senescence symptoms on d 3, such as drooped (*side view*) and bluing petals (*top view*)

Next, we compared the morphological changes of *RhMKK9*-silenced flowers with the TRV controls. On the first day after rehydration, petal loosening of the inner whorls of the TRV control was more obvious than that in the *RhMKK9*-silenced flowers, implying that *RhMKK9* silencing delayed the flower opening process. On the third day after rehydration, petals of the TRV2 flowers, especially in the inner whorl, faded to a bluish colour and the petals tended to droop in side view, indicating the flowers entered the phase of senescence (Fig. 4a, bottom panel) [23–25]. However, suppression of *RhMKK9* expression obviously inhibited the petal bluing and drooping, implying that petal senescence was delayed (Fig. 4a, top panel). We also detected ethylene production in gynoecia of the TRV control and *RhMKK9-*silenced flowers, respectively, during the dehydration and rehydration process. Similarly to the untreated flowers, ethylene production was triggered rapidly by rehydration in gynoecia of TRV control flowers [16] (Fig. 4b). However, silencing of *RhMKK9* significantly decreased the rehydration-induced ethylene production in gynoecia, indicating that *RhMKK9* was required for the rehydration-caused increase of ethylene production in gynoecia (Fig. 4b).

### Silencing of *RhMKK9* downregulated the expression of genes associated with senescence and induced by ethylene in petals

**Fig. 5:**
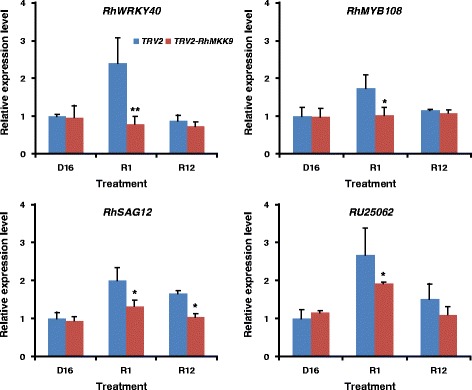
Expression patterns of four ethylene-responsive genes in petals of TRV control and RhMKK9-silenced flowers. At least three biological replicates were tested for each time point. *Asterisks* in the bottom panel indicate significant differences calculated using the *t* test (**p < 0.01, *p < 0.05). D16, dehydration for 16 h; R1 and R12, rehydration for 1 h and 12 h, respectively

Previous reports showed that rehydration-caused ethylene production in rose gynoecia could accelerate petal senescence by regulating the expression of genes downstream to ethylene signalling [7]. To obtain further insights into *RhMKK9-*silencing-delayed petal senescence, we monitored the expression of four genes in petals of TRV control and *RhMKK9*-silenced flowers during dehydration and rehydration. These four tested genes were all ethylene-induced and included two transcription factor genes involving abiotic response and senescence, *RhWRKY40* and *RhMYB108* [22], a senescence-associated marker gene *RhSAG12* [26], as well as an unknown gene, which was highly induced by ethylene [22]. We found that the expression of these four genes was significantly reduced in petals of *RhMKK9*-silenced flowers in the first hour of rehydration than those of the TRV controls (Fig. 5). During the twelfth hour after rehydration, *RhWRKY40* and *RhMYB108* expression in TRV control flowers was decreased to a relatively low level, which was similar to that of *RhMKK9*-silenced flowers, possibly owing to the low ethylene production level at this time (Fig. 5). However, *RhSAG12* expression was still significantly higher in TRV controls than in *RhMKK9*-silenced flowers, indicating that the senescence process of TRV control petals might be accelerated. These results implied that rehydration-caused ethylene production, which was a required action of *RhMKK9*, promoted the transcript accumulation of genes related to petal senescence and resulted in earlier petal senescence.

### DNA methylation status of the *RhMKK9* promoter and gene body is altered during rehydration

**Fig. 6:**
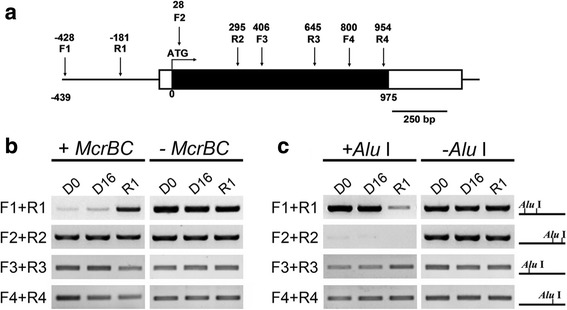
Effects of dehydration and rehydration on DNA methylation of the *RhMKK9* gene. The effects of dehydration and rehydration on cytosine DNA methylation in *RhMKK9* gene (**a**) were determined by *McrBC* digestion (**b**) and *Alu* I-mediated (**c**) Chop-PCR assays. **a** Schematic structure of *RhMKK9* gene. The primers used for the Chop-PCR assay are indicated as F1 + R1, F2 + R2, F3 + R3, F4 + R4, and F5 + R5. **b** *McrBC* digestion assay. Genomic DNA was digested with *McrBC* for 3 h and amplified by PCR. **c** *Alu* I-mediated Chop-PCR assay. Linearised genomic DNA was digested with *Alu* I for 3 h, respectively, and then was amplified by PCR. The right panel indicates the *Alu* I site in each tested region. In each assay, undigested genomic DNA was used as a control. Primers were listed in Additional file 1: Table S2. D0 and D16, dehydration for 0 h and 16 h, respectively; R1, rehydration for 1 h. Note, there is no intron in *RhMKK9*

Epigenetic modification has been reported to regulate multiple tolerances of plants to environmental stresses, including heat stress, cold, and dehydration [27–30]. Recently, a report showed that histone modification was associated with inactivation of dehydration-inducible genes by rehydration in *Arabidopsis* [6]. Here, we tested the DNA methylation status of the *RhMKK9* promoter and gene body by DNA methylation sensitive Chop-PCR. Five pairs of primers were designed to cover the ~500 bp upstream region to RhMKK9 ORF and gene body (Fig. 6a, Additional file 1: Figure S2). DNA methylation-specific *McrBC* digestion showed that the F1 + R1 region was heavily methylated during dehydration, whereas they were de-methylated when the flowers were subjected to rehydration (Fig. 6b). However, the DNA methylation level of *RhMKK9* ORF, including the F2 + R2, F3 + R3, and F4 + R4 regions, was slightly elevated during rehydration (Fig. 6b). DNA methylation was further tested by digestion with *Alu* I. The results further supported that the F1 + R1 region of the *RhMKK9* promoter was hypomethylated, whereas the *RhMKK9* gene body was hypermethylated during rehydration (Fig. 6c). In general, DNA methylation on the promoter region was considered to play an inhibitory role in gene transcription, whereas DNA methylation on the gene body indicated the activation of gene expression [31, 32]. Thus, the results of the DNA methylation assay further supported the gene expression analysis of *RhMKK9* during dehydration and rehydration, revealing that the induction of *RhMKK9* expression by rehydration might result from rehydration-caused DNA demethylation in the *RhMKK9* promoter.

## Discussion

Despite its simple structure, ethylene is functionally complex and plays a crucial role in a broad spectrum of plant developmental and environmental responses [33, 34]. Ethylene acts as an essential mediator in post-pollination fertilisation and ovule development, as well as associated petal senescence in various plants [35–39]. In *Arabidopsis*, ethylene was reported to be indispensable for fertilisation by inducing synergid cell death and establishing pollen tube block [39]. In *Phalaenopsis*, a pollination-induced ethylene burst in the stigma and style ensured appropriate ovary development, and considerably promoted perianth senescence [35, 37]. Similarly, in carnation (*Dianthus caryophyllus*) flowers, removal of gynoecia repressed the production of ethylene and delayed petal senescence [40].

Among the various abiotic stresses, dehydration is known to influence ethylene production in plants, although the underlying regulatory mechanism seems complex. In plants such as cotton (*Gossypium hirsutum* L.) petioles and bolls [41, 42], wheat (*Triticum aestivum* L.) leaves [43], orange (*Citrus sinensis* Osbeck) flowers [44], as well as persimmon (*Diospyros kaki* Thunb.), and avocado (*Persea americana* Mill.) fruit, dehydration induces ethylene production [45, 46]. In detached persimmon fruit, water-loss induced ethylene burst in the calyx triggered ethylene production in other tissues and accelerated fruit ripening and softening [46].

In rose flowers, dehydration gradually increased ethylene production in sepals, whereas rehydration following a period of dehydration triggered rapid ethylene production in gynoecia [7]. Moreover, the ethylene production in gynoecia, although very transient, is essential for the flower to fully open, suggesting that ethylene is naturally required for the water recovery of dehydrated flowers [16]. Considering that continuous ethylene production is very harmful to flowers, the timing of ethylene production should be tightly regulated. Biochemical analysis demonstrated that protein phosphorylation-dependent accumulation of RhACS1, a rate-limiting enzyme of ethylene biosynthesis, is responsible for controlling the timing of ethylene production. In addition, RhACS1 protein phosphorylation is attributed to RhMPK6, a MAP kinase, which is also precisely regulated by rehydration in rose flowers [16]. More important, RhMPK6 abundance is retained at a relatively constant level during both dehydration and rehydration, whereas RhMPK6 activation only occurs within 1 h of rehydration. Thus, these results indicate that appropriate ethylene production is important for the natural recovery process of rose flowers during rehydration. Therefore, it is not necessary for constant RhMPK6 accumulation during dehydration and rehydration, which is an energy-consuming process. Moreover, because MAPKs must be activated by MAPK KINASEs, it is reasonable to speculate that an upstream component should be involved in the rehydration-activation of RhMPK6.

In *Arabidopsis*, MPK6 has been reported to be activated by different MAPK kinases in response to various biotic and abiotic stresses [47, 48]. Cold and salinity stress caused activation of MPK6 is, at least partially, dependent on MKK2 [49, 50], whereas MKK5 is required for oxidative-triggered MPK6 [51, 52]. For drought stress, although MPK6 is activated by drought-induced ROS accumulation [53], the corresponding MAPK kinase has not been identified. Expression of the active version of MKK9 promotes MPK3 and MPK6 kinase activity and ethylene production, and enhances the sensitivity of transgenic *Arabidopsis* to salt stress [54].

Here, we isolated the homologues of *MKK2*, *MKK4*, *MKK5*, and *MKK9* from roses and detected their expression during dehydration and rehydration. It is noteworthy that *RhMKK9* expression, which could phosphorylate RhMPK6 in vitro [16], was relatively lower during dehydration, but significantly and sharply induced at the onset of rehydration, and then rapidly decreased to similar level before rehydration. Furthermore, specific silencing of *RhMKK9* led to a significant reduction of rehydration-induced ethylene production in gynoecia. The rehydration-responsive expression pattern and function identification suggested that *RhMKK9* is possibly the upstream component regulating the rehydration-activated RhMPK6-RhACS1 cascade and ethylene production in rose gynoecia. Interestingly, *RhMKK9* silencing delayed both flower opening and senescence, further supporting that rehydration-caused ethylene production accelerated flower opening and senescence. Detection of gene expression showed that rehydration led to the rapid expression elevation of several ethylene-inducible genes, which were involved in abiotic stress-response and senescence, in TRV control flowers. Consistently, expression of ethylene-inducible genes was significantly inhibited in *RhMKK9*-silenced flowers. It is worth noting that the expression of two transcription factor genes, *RhWRKY40* and *RhMYB108*, decreased after 12 h of rehydration in TRV controls to a relatively low level, similarly to *RhMKK9*-silenced flowers. Therefore, *RhWRKY40* and *RhMYB108* might be closely associated with ethylene production. However, expression of the senescence-associated *RhSAG12* gene in the TRV control was significantly higher than that in *RhMKK9*-silenced flowers at 12 h after rehydration, implying that rehydration-caused ethylene production might also initiate the petal senescence process.

In the last decade, increasing evidence has shown epigenetic modification plays crucial roles in tolerance, adaption, and memory of plants to various abiotic stresses [27–30]. In *Arabidopsis*, histone modification of H3K4me3 and H3K9ac were enriched in drought stress-induced genes [6, 30, 55]. In the rehydration process, H3K9ac was rapidly removed, correlating to the inactivation of drought-inducible genes. Interestingly, H3K4me3 was removed more slowly than the H3K9ac mark, suggesting that H3K4me3 may be responsible for the epigenetic memory of drought [6, 30]. In rice, drought stress induced gene expression of the histone acetyltransferase (HATs) family and enhanced acetylation of H3K9, H3K18, H3K27, and H4K5 [56].

Dynamic DNA methylation and demethylation has also been broadly reported to be involved in plant responses to abiotic stresses [57–61]. In maize, cold stress-induced *ZmMI1* gene expression is associated with DNA hypomethylation in the nucleosome cores [62]. Here, we found that rehydration also resulted in hypomethylation on the *RhMKK9* promoter, whereas elevated methylation on the *RhMKK9* gene body. The factors involved in this rapid methylation and de-methylation process should be the subject of further studies.

## Conclusions

In summary, a MAPK KINASE, RhMKK9, is the upstream component responsible for activating the RhMPK6-RhACS1 cascade. *RhMKK9* expression was specifically and rapidly induced by rehydration in gynoecia. *RhMKK9* silencing inhibited rehydration-caused ethylene bursts and delayed flower opening and senescence. In addition, we found that changes of DNA methylation status on the *RhMKK9* promoter and gene body were associated with *RhMKK9* induction by rehydration. These results explained how the flower, the reproductive organ, could quickly recover from dehydration using ethylene as a mediator.

## Methods

### Plant materials

Flowers of *R. hybrida* ‘Samantha’ were provided by a commercial greenhouse (Sunstone Company) in the Changping District, Beijing. The flowers were harvested at opening stage 2 as described previously [18]. The flowers were immediately placed in tap water and transported to the laboratory within 1 h. Stems of the rose flowers were re-cut underwater to approximately 25 cm, and then were kept in deionised water (DW) until further processing.

### Dehydration and rehydration treatments

Dehydration treatment was conducted as described previously [7, 16]. Flowers were placed horizontally on the bench in a climate-controlled room at 25 °C, 40–50% relative humidity, and a continuous light with intensity of 140 μmol m^−2^ s^−1^. The dehydration status was defined by fresh weight loss, and the flowers were subjected to rehydration when the flowers lost ~30% fresh weight. After the dehydration treatment, the bottom of flower stems were re-cut to remove about 1 cm under water to prevent air embolisms, and the flowers were placed in deionised water for rehydration. The flower phenotype was observed at different time points: 0, 1, 2, and 3 d after rehydration. After treatment, the flowers were cut open and the gynoecia, including carpels and pistils, were sampled as described previously [7, 16, 20]. Half of the gynoecia at D16 (dehydration for 16 h) and R1 (rehydration for 1 h) were taken for the ethylene production test, whereas the petals and the other half of the gynoecia were collected and frozen using liquid nitrogen and then were stored at −80 °C for RNA isolation.

### Ethylene production measurement

The gynoecia were weighed and then placed in a 25 ml airtight GC vial, and the vials were kept for 30 min at 25 °C. From each vial, 2 μl headspace gas was withdrawn to measure ethylene concentration using a gas chromatograph equipped with a flame ionisation detector (GC-17A, Shimadzu, Japan) as described previously [7, 20]. After ethylene measurement, the gynoecia were dried in an oven at 60 °C to determine their dry weight, and then the ethylene production was calculated. Fifteen flowers were used for each time point.

### Sequence analysis

Alignment of multiple deduced amino acid sequences was constructed using ClustalW2 software and visualised using the BioEdit program. Phylogenetic analysis was performed using MEGA 5.2.

### RNA extraction, semi-quantitative RT-PCR analyses

Total RNA of petals was isolated using the hot borate method as described previously [19], and the total RNA of gynoecia was extracted using a RN38-EASY RNA extraction kit (Aidlab, Co, Ltd., Beijing, PRC). Total RNA was treated by RNase-free DNase I to avoid genomic DNA contamination. An volume of 1 μg of clean RNA was used to synthesise cDNA using M-MLV reverse transcriptase (Promega Corp., Madison, WI, USA) according to the manufacturer’s instructions. The rose ubiquitin gene (*RhUBI*, JK622648) was used as the internal control. The primers used in the RT-PCR analysis are listed in Additional file 1: Table S2. PCR reactions were carried out for 5 min at 94 °C, followed by 30 cycles of 30 s at 94 °C, 30 s at 58 °C, 30 s at 72 °C, and followed by a supplemental incubation for 7 min at 72 °C for all the genes. The PCR products were separated on 1.5% agarose gels and visualised by ethidium bromide staining. Absolute values for transcript abundance from RT-PCR were quantified using the Alpha EaseFCTM 2200 software (Alpha Innotech, USA, Version 3.2.1). For all experiments, an individual flower was considered as a biological replicate and all experiments were performed with at least three replicates.

*RhMKK9* gene silencing was performed as previously described [19]. A 490-bp fragment from the *RhMKK9*-specific 3′ end (partial ORF and entire 3′UTR) was used to construct pTRV2-*RhMKK9*. The resulting constructs pTRV2-*RhMKK9*, pTRV1, and pTRV2 were transformed into *Agrobacterium tumefaciens* strain GV3101. Briefly, *Agrobacterium* was grown in LB broth containing 50 μg ml^−1^ kanamycin and 50 μg ml^−1^ gentamycin sulphate at 28 °C with shaking at 200 rpm overnight. These cultures were then diluted 1:50 v/v in fresh LB broth containing 10 mM MES, 20 mM acetosyringone, 50 μg ml^−1^ kanamycin, and 50 μg ml^−1^ gentamycin sulphate, and grown overnight as described above. *Agrobacterium* cells were harvested by centrifugation, and the pellet was suspended in infiltration buffer (10 mM MgCl_2_, 150 mM acetosyringone, and 10 mM MES, pH 5.6) to a final A600 of 1.8. A mixture of *Agrobacterium* cultures containing pTRV1 and pTRV2-*RhMKK9* at a ratio of 1:1 (v/v) was used for rose transformation, and a mixture containing pTRV1 and pTRV2 was used as the negative control. The mixtures were stored at room temperature for 4 h in the dark prior to vacuum infiltration.

For vacuum infiltration, the flowers were placed upside down in a container (81.64 L), with the whole flower immersed into the prepared infiltration mixture. The flowers were then infiltrated by vacuum at 30 mmHg twice, each for 2 min. Then they were briefly washed with DW and kept in DW for 3 d at 8 °C before the dehydration treatment [16].

### Immunoblot and kinase activity assays

Gynoecia protein was extracted as described previously [63]. The following antibodies were used: goat polyclonal IgG anti-ACC synthase 6 antibody (SC-12771, Santa Cruz Biotechnology, http://www.scbt.com) to detect RhACS1; polyclonal anti-MPK6 (Sigma-Aldrich, http://www.sigmaaldrich.com) to detect RhMPK6. Secondary antibodies (horseradish peroxidase-conjugated goat anti-mouse IgG, Sigma-Aldrich) were used as recommended in the manufacturer’s instructions.

For the kinase activity assay, SDS-PAGE gel was supplied with 25 μM Phos-tag (Wako Chemicals) and 50 μM Zn^+^ was used as described previously [64–66]. The running buffer contained 100 mM Tris, 100 mM MOPS, and 0.1% (w/v) SDS. Sodium bisulphite was added to 5 mM immediately before electrophoresis.

### DNA methylation assay

DNA methylation status was analysed by Chop-PCR as described previously [67]. For Chop-PCR, genomic DNA (500 ng) was digested with the methylation-sensitive restriction enzyme *Alu* I, or by the methylated DNA-digesting enzyme *McrBC* for 3 h. The digested DNA was used as a template to amplify the *RhMKK9* promoter and gene body. Undigested genomic DNA was amplified as an internal control.

## Additional file

Additional file 1: Figure S1. Alignment analysis of RhMKK2 (A), RhMKK4 (B), and RhMKK5 (C). The proteins used include, RhMKK2, rose (*Rose hybrida*), ALG02503.1; FvMKK2, woodland strawberry (*Fragaria vesca* subsp*. vesca*), XP_011460401.1; MdMKK2, apple (*Malus domestica*), XP_008337740.1; PbMKK2, Chinese white pear (*Pyrus* x *bretschneideri*), XP_009361655.1; PmMKK2, Chinese plum (*Prunus mume*), XP_008242181.1; EgMKK2, rose gum (*Eucalyptus grandis*), XP_010047526.1; RhMKK4, rose (*R. hybrida*), ALG02504.1; MdMKK5, apple (*M. domestica*), XP_008380261.1; FvMKK5, woodland strawberry (*F. vesca subsp. vesca*), XP_004303847.1; PbMKK5, Chinese white pear (*P.* x *bretschneideri*), XP_009333993.1; PmMKK5, Chinese plum (*Prunus mume*), XP_008229371.1; PtMKK4, black cottonwood (*Populus trichocarpa*); RhMKK5, rose (*R. hybrida*), XP_006379405.1; ALG02505.1. **Table S1.** Gene information of rose MAP KINASE KINASE. **Table S2.** Oligonucleotide primer sequences. (DOC 384 kb)

## Abbreviations

ACS: 1-aminocyclopropane-1-carboxylic acid synthase
MAPK: Mitogen-activated protein kinase
MAPKKK: Mitogen-activated protein kinase kinase kinase
MKK: Mitogen-activated protein kinase kinase
ORF: Open reading frame
SAG: Senescence-associated gene
TRV: Tobacco rattle virus
UTR: Untranslated region
VIGS: Virus-induced gene silencing
