# Supplementary material for: RhMKK9, a rose MAP KINASE KINASE gene, is involved in rehydration-triggered ethylene production in rose gynoecia
Source: BMC Plant Biol. 2017 Feb 23;17:51. doi: 10.1186/s12870-017-0999-1 (PMC5322680; doi:10.1186/s12870-017-0999-1)
Supplement: Additional file 1: Figure S1. — Alignment analysis of RhMKK2 (A), RhMKK4 (B), and RhMKK5 (C). The proteins used include, RhMKK2, rose (Rose hybrida), ALG02503.1; FvMKK2, woodland strawberry (Fragaria vesca subsp. vesca), XP_011460401.1; MdMKK2, apple (Malus domestica), XP_008337740.1; PbMKK2, Chinese white pear (Pyrus x bretschneideri), XP_009361655.1; PmMKK2, Chinese plum (Prunus mume), XP_008242181.1; EgMKK2, rose gum (Eucalyptus grandis), XP_010047526.1; RhMKK4, rose (R. hybrida), ALG02504.1; MdMKK5, apple (M. domestica), XP_008380261.1; FvMKK5, woodland strawberry (F. vesca subsp. vesca), XP_004303847.1; PbMKK5, Chinese white pear (P. x bretschneideri), XP_009333993.1; PmMKK5, Chinese plum (Prunus mume), XP_008229371.1; PtMKK4, black cottonwood (Populus trichocarpa); RhMKK5, rose (R. hybrida), XP_006379405.1; ALG02505.1. Table S1. Gene information of rose MAP KINASE KINASE. Table S2. Oligonucleotide primer sequences. (DOC 384 kb) [file 12870_2017_999_MOESM1_ESM.doc]

**A**


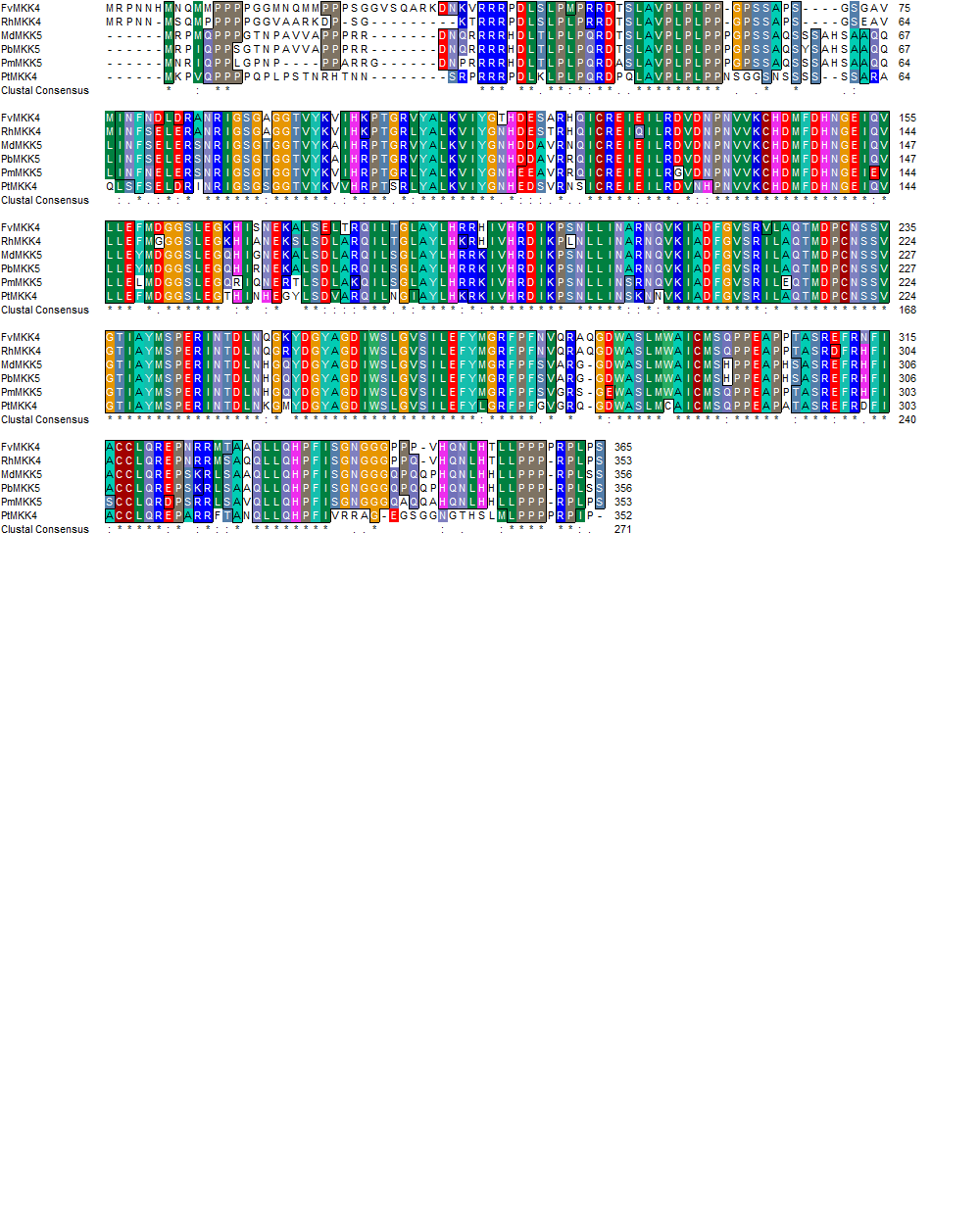


**B**

**C**


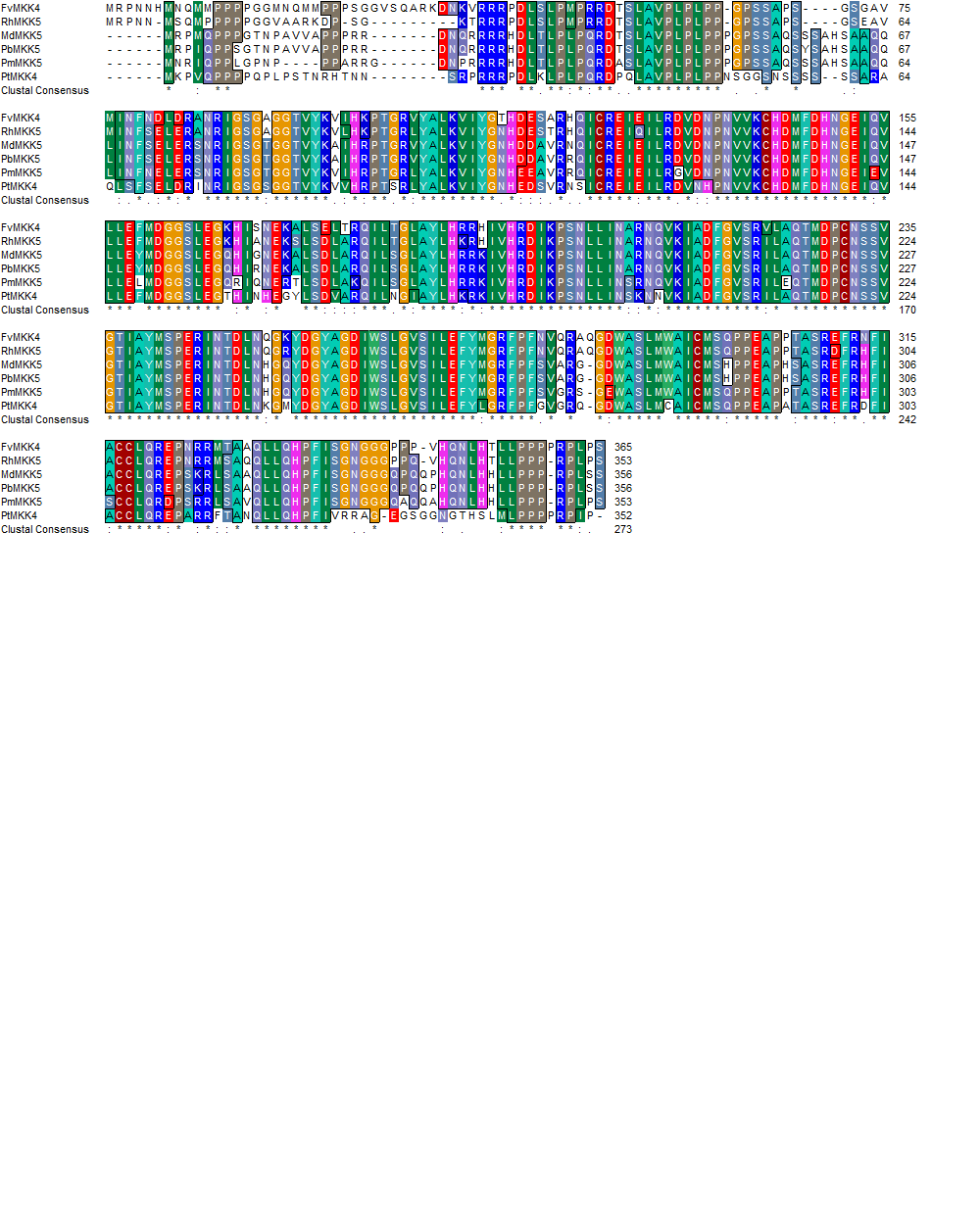


**Fig. S1 Alignment analysis of RhMKK2 (A), RhMKK4 (B) and RhMKK5 (C).**

| Supplemental Table 1. Gene information of rose MAP KINASE KINASE. | | | | | |
| --- | --- | --- | --- | --- | --- |
| Gene Name | Accession Number | Full length (bp) | ORF (bp) | 5'-UTR (bp) | 3'-UTR (bp) |
| *RhMKK2* | KP269070.1 | 1,453 | 1,065 | 108 | 280 |
| *RhMKK4* | KP269071.1 | 1,522 | 1,062 | 130 | 330 |
| *RhMKK5* | KP269072.1 | 1,508 | 1,062 | 337 | 109 |
| *RhMKK9* | KP269073.1 | 1,367 | 975 | 59 | 333 |

| **Supplemental Table 2**. Oligonucleotide primer sequences | | | |
| --- | --- | --- | --- |
| ID | Sequence | Purpose | Amplicon length (bp) |
| *RhUBI* FWD | 5’-CGGTTTCTGGATGAAGGAAAG-3’ | RT-PCR | 452 |
| *RhUBI* REV | 5’-TTGAAAGGCAGTCGTCTTGG-3’ | RT-PCR |  |
| *RhMKK9 F*WD | 5’-CACAGTCTACAAAGTCCGCC-3’ | RT-PCR | 426 |
| *RhMKK9* REV | 5’-GTTATCGAGAGTTCGGCACA-3’ | RT-PCR |  |
| *RhMKK2*FWD | 5’-TGTTGTTGACTTGCCACCACCT-3’ | RT-PCR | 383 |
| *RhMKK2 REV* | 5’-CTCGCTACCAAAGGGAACTAACA-3’ | RT-PCR |  |
| *RhMKK4* FWD | 5’-TCGTCAAGTGCCACGATATG-3’ | RT-PCR | 425 |
| *RhMKK4* REV | 5’-CACATTGAACGGAAATCGGC-3’ | RT-PCR |  |
| *RhMKK5* FWD | 5’-CCCGTCGGGGAAGACAC-3’ | RT-PCR | 299 |
| *RhMKK5* REV | 5’-CGGAGGATTTGGATCTCGC-3’ | RT-PCR |  |
| *RhSAG12* FWD | 5'-ACCAGAGAACACCCCCACTACT-3' | RT-PCR | 303 |
| *RhSAG12* REV | 5'-AACTCGTGGATTGTGATGATGG-3' | RT-PCR |  |
| *RhWRKY40* FWD | 5’-ATAGCTCAATCGATAACCAAGCA-3’ | RT-PCR | 431 |
| *RhWRKY40* REV | 5’-TCAACCTCTCTTGGCACATC-3’ | RT-PCR |  |
| *RhMYB108* FWD | 5’-GTGGCAACATCACTCTCGAA -3’ | RT-PCR | 379 |
| *RhMYB108* REV | 5’-GTTGCCTGTTGTAAAGCGAC-3’ | RT-PCR |  |
| *RU25062* FWD | 5’-TAACACTCCCCTTTTATCTCC-3’ | RT-PCR | 320 |
| *RU25062* REV | 5’TTTTAGGTACTTCACGGTCG-3’ | RT-PCR |  |
| *RNA1* FWD | 5'-TTACAGGTTATTTGGGCTAG-3' | RT-PCR | 647 |
| *RNA1* REV | 5'-CCGGGTTCAATTCCTTATC-3' | RT-PCR |  |
| *RNA2* FWD | 5'-TGGGAGATGATACGCTGTT-3' | RT-PCR | 281 |
| *RNA2* REV | 5'-CCTAAAACTTCAGACACG-3' | RT-PCR |  |
| F1 | 5-CAGAGGGAGTTGGCGGAGAC-3 | Chop-PCR | 248 |
| R1 | 5'-CGCTTCACTTCAGCCATTCG-3' | Chop-PCR |  |
| F2 | 5'-TCAACCTCAGCCTCCCC-3' | Chop-PCR | 269 |
| R2 | 5'-TCTATTTCGCGGTAGAGCTGG-3' | Chop-PCR |  |
| F3 | 5'-AGACCTCGCTCAAAGAAAACG-3' | Chop-PCR | 240 |
| R3 | 5'-TCATGTAAGCACAAGTCCCAA-3' | Chop-PCR |  |
| F4 | 5'-CGCCATTTGTTTCGGGGAG-3' | Chop-PCR | 154 |
| R4 | 5'-TGGCTTTCGGATCTTTACAAAC-3' | Chop-PCR |  |
| RNA2-*RhMKK9* FWD | 5'-GCCTCTAGAAGCCACCGGCCTTGCC-3' | VIGS | 491 |
| RNA2-*RhMKK9* REV | 5'-AATCTCGAGGATTAAATATGAAACTCTTTC-3' | VIGS |  |
